# Correction to: SENP3‐mediated TIP60 deSUMOylation is required for DNA‐PKcs activity and DNA damage repair

**DOI:** 10.1002/mco2.197

**Published:** 2022-12-01

Yang Han, Xin Huang, Xiaoyu Cao, Yuchen Li, Lei Gao, Jin Jia,Gang Li, Hejiang Guo, Xiaochang Liu, Hongling Zhao, Hua Guan, Pingkun Zhou,Shanshan Gao

Correction to: *MedComm*  https://doi.org/10.1002/mco2.123, published online 22 March 2022

In the process of checking the raw data,^1^ the authors noticed several inadvertent mistakes occurring in Fig. 1E and Fig. 3A that need to be corrected after online publication of the article. During the preparation of Fig. 1E and Fig. 3A, the representative image showing the expression of TIP60 in the input panel and the representative image showing the localization of TIP60 in the NR panel, were pasted and placed by mistake. The correct results should be as shown below. The authors apologize for these oversights and declare that these corrections do not affect the description, interpretation, or conclusions detailed in the original manuscript.

**FIGURE 1:**
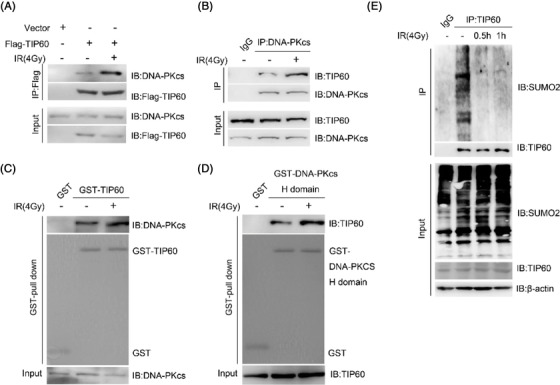
The interaction between DNA‐PKcs and TIP60 is increased upon irradiation‐induced DNA damage. **(A)** After transitory transfection using Flag‐tagged TIP60, the HeLA cells were subjected to treatment either with or without 4 Gy γ‐ray irradiation, 1 h later, anti‐Flag affinity gel was used to accomplish immunoprecipitation of the harvested cell lysates, and designated DNA‐PKcs antibody was used to conduct western blot. **(B)** The TIP60–DNA‐PKc interplay in non‐radiated and 1 h radiated (4 Gy) HeLA cells was validated through co‐immunoprecipitation (Co‐IP) assays using either anti‐Flag or DNA‐PKcs antibody. The Co‐IP samples were subjected to SDS‐PAGE isolation and subsequent immunoblotting for the designated proteins. **(C)** *E. coli* (BL21) bacterial expression of TIP60 based on the GST‐pull‐down assay. The pull‐down productions were western blotted using the DNA‐PKcs antibody. **(D)** *E. coli* (BL21) bacterial expression of DNA‐PKcs H domain (AA3540‐4128) based on the GST‐pull‐down assay. The western blotting of pull‐down productions was performed with the TIP60 antibody. **(E)** After transitory transfection using designated plasmids, the HEK‐293T cells were subjected to treatment under 4 Gy γ‐ray irradiation, followed by collection and separate 0.5‐ and 1‐h lysing treatments. Flag beads were utilised to pull down the SUMOylated TIP60 proteins, which were then examined through Western blot.

**FIGURE 3:**
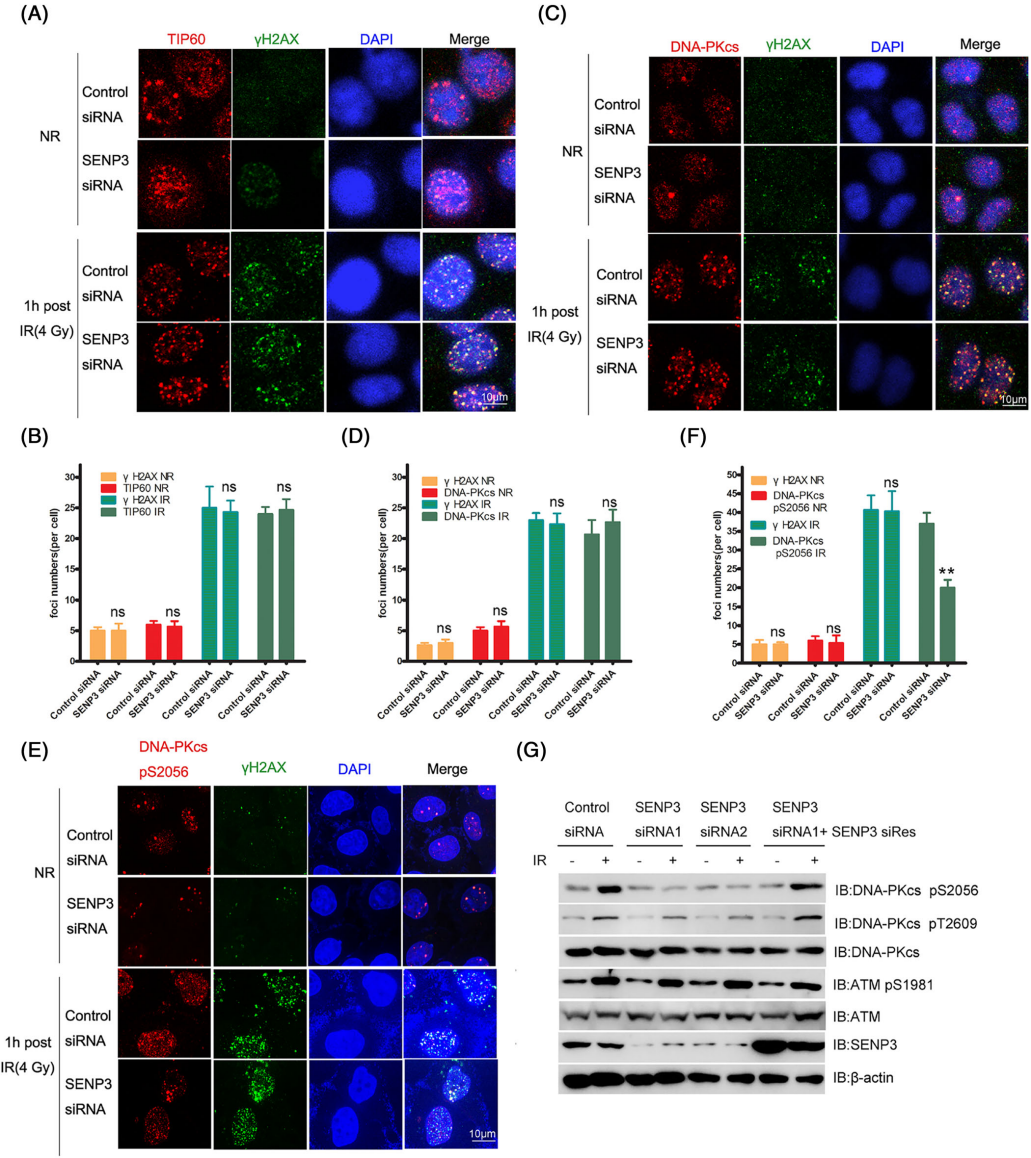
Knocking down of SENP3 decreases the DNA‐PKcs activity upon DNA damage. **(A)** Twenty‐four hours following transfection using SENP3 siRNA or control, the HeLA cells were irradiated with or without 4Gy γ‐ray. The TIP60 and γH2AX expressions were examined 1 h later, which was accomplished through immunofluorescence assay using corresponding antibodies. **(B)** Quantification of the foci numbers of γH2AX and TIP60 in the HeLA cells post 4 Gy γ‐ray irradiation. Data were represented as means ± SDs of triplicate (at least) experiments. In every experiment, scoring was made on 50 cells. ns means no significance. **(C)** Twenty‐four hours following transfection using SENP3 siRNA or control, the HeLA cells were subjected to treatment with or without 4 Gy γ‐ray irradiation. The DNA‐PKcs and γH2AX expressions were examined 1 h later, which was accomplished through immunofluorescence assay with corresponding antibodies. **(D)** Quantification of the foci numbers of γH2AX and DNA‐PKcs in the HeLA cells post 4 Gy γ‐ray irradiation. Data were represented as means ± SDs of triplicate (at least) experiments. In every experiment, scoring was made on 50 cells. ns means no significance. **(E)** Twenty‐four hours following transfection using SENP3 siRNA or control, the HeLA cells were subjected to treatment with or without 4 Gy γ‐ray irradiation. One hour later, immunofluorescence assay was performed to test the expression of DNA‐PKcs pS2056 and γH2AX using corresponding antibodies. **(F)** Quantification of the foci numbers of γH2AX and DNA‐PKcs pS2056 in the HeLA cells post 4 Gy γ‐ray irradiation. Data were represented as means ± SDs of triplicate (at least) experiments. In every experiment, scoring was made on 50 cells. ns means no significance,**p < 0.01. **(G)** Knocking down SENP3 using two single siRNA against SENP3 (SENP3 siRNA1 and siRNA2) or Control siRNA in HeLA cells, and re‐expressed SENP3 using SENP3 siRes. Subsequently, treatment of cells proceeded in the presence or absence of irradiation with 4 Gy γ‐ray. The resulting cells were then harvested and lysed at 1 h after irradiation. SDS‐PAGE was conducted for sample isolation, while sample incubation was accomplished using designated antibodies.

## References

[1] Yang H , Xin H , Xiaoyu C et al., SENP3‐mediated TIP60 deSUMOylation is required for DNA‐PKcs activity and DNA damage repair. MedComm. 2022 Mar 22;3(2):e123. 10.1002/mco2.123 35356800 PMC8941250

